# Osteopontin: A Key Regulator of Tumor Progression and Immunomodulation

**DOI:** 10.3390/cancers12113379

**Published:** 2020-11-15

**Authors:** Hannah R. Moorman, Dakota Poschel, John D. Klement, Chunwan Lu, Priscilla S. Redd, Kebin Liu

**Affiliations:** 1 Department of Biochemistry and Molecular Biology, Medical College of Georgia, Augusta, GA 30912, USA; Hmoorman@augusta.edu (H.R.M.); Dposchel@augusta.edu (D.P.); Jklement@augusta.edu (J.D.K.); Clu@augusta.edu (C.L.); 2 Georgia Cancer Center, Medical College of Georgia, Augusta, GA 30912, USA; 3 Charlie Norwood VA Medical Center, Augusta, GA 30904, USA; 4 Chemedimmune Inc., Augusta, GA 30912, USA; Predd@augusta.edu

**Keywords:** osteopontin, CD44, integrin, tumor-associated macrophage, MDSCs, immune evasion, immune checkpoint

## Abstract

**Simple Summary:**

Anti-PD-1/PD-L1 and anti-CTLA-4-based immune checkpoint blockade (ICB) immunotherapy have recently emerged as a breakthrough in human cancer treatment. Durable efficacy has been achieved in many types of human cancers. However, not all human cancers respond to current ICB immunotherapy and only a fraction of the responsive cancers exhibit efficacy. Osteopontin (OPN) expression is highly elevated in human cancers and functions as a tumor promoter. Emerging data suggest that OPN may also regulate immune cell function in the tumor microenvironment. This review aims at OPN function in human cancer progression and new findings of OPN as a new immune checkpoint. We propose that OPN compensates PD-L1 function to promote tumor immune evasion, which may underlie human cancer non-response to current ICB immunotherapy.

**Abstract:**

OPN is a multifunctional phosphoglycoprotein expressed in a wide range of cells, including osteoclasts, osteoblasts, neurons, epithelial cells, T, B, NK, NK T, myeloid, and innate lymphoid cells. OPN plays an important role in diverse biological processes and is implicated in multiple diseases such as cardiovascular, diabetes, kidney, proinflammatory, fibrosis, nephrolithiasis, wound healing, and cancer. In cancer patients, overexpressed OPN is often detected in the tumor microenvironment and elevated serum OPN level is correlated with poor prognosis. Initially identified in activated T cells and termed as early T cell activation gene, OPN links innate cells to adaptive cells in immune response to infection and cancer. Recent single cell RNA sequencing revealed that OPN is primarily expressed in tumor cells and tumor-infiltrating myeloid cells in human cancer patients. Emerging experimental data reveal a key role of OPN is tumor immune evasion through regulating macrophage polarization, recruitment, and inhibition of T cell activation in the tumor microenvironment. Therefore, in addition to its well-established direct tumor cell promotion function, OPN also acts as an immune checkpoint to negatively regulate T cell activation. The OPN protein level is highly elevated in peripheral blood of human cancer patients. OPN blockade immunotherapy with OPN neutralization monoclonal antibodies (mAbs) thus represents an attractive approach in human cancer immunotherapy.

## 1. Introduction

Osteopontin (OPN), a phosphorylated glycoprotein, was originally identified as a secreted form in bone and later discovered as an intracellular protein [1,2]. OPN is widely expressed in osteoclasts; osteoblasts; epithelial cells of the breast, kidney, and skin; nerve cells; vascular smooth muscle cells; endothelial cells; and fibroblasts [3,4]. A broad range of immune cells such as macrophages, lymphocytes, natural killer cells, eosinophils, dendritic cells, and microglia express OPN [5,6,7,8,9,10,11]. Given OPN’s wide expression profile, it follows that OPN is not only important for normal biological functioning such as bone remodeling, immunity, and inflammation, but also plays a role in pathological processes such as liver fibrosis, atherosclerosis, and cancer [12,13].

In cancer patients, OPN is often overexpressed in cells of the tumor microenvironment and elevated in the peripheral blood. OPN overexpression has been associated with worse prognosis in many cancers including glioblastoma multiforme, hepatocellular carcinoma, colorectal cancer, lung cancer, breast cancer, bladder cancer, melanoma, head and neck squamous cell carcinoma, and acute myeloid leukemia [14,15,16,17,18,19,20,21,22,23,24,25,26,27,28,29,30,31,32,33,34,35,36,37,38,39]. OPN contributes to the malignancy of cancer through the promotion of metastasis, maintenance of a stem-like phenotype, epithelial to mesenchymal transformation, activation of cell proliferation pathways, chemotherapeutic and radiation resistance, and interference with immune functioning. As such, understanding OPN’s regulation and mechanisms of action will provide potential treatment options in the future. In this review, we attempt to address the role of OPN in cancers including clinical data, mechanisms, regulation, and potential treatment strategies.

## 2. The OPN Protein

**Figure 1:**
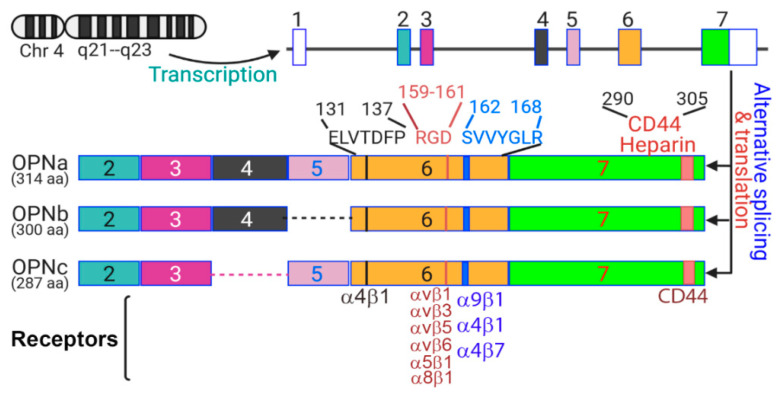
Human OPN protein structures. The human SPP1 genes that encodes the OPN protein has seven exons. Three alternative spliced OPN variants translate to protein. The receptor-interacting domains are shown. The respective receptors for these domains are shown at the bottom.

OPN is a member of the SIBLING (Small Integrin-Binding Ligand, N-linked Glycoprotein) family of proteins [40]. OPN, also known as secreted phosphoprotein 1, is encoded by a single-copy gene, *spp1*, on human chromosome 4 near the centromere [41]. It has several conserved structural domains including an RGD domain (arginine-glycine-aspartate), SVVYGLR domain (Serine-valine-valine-tyrosine-glutamate-leucine-arginine), heparin-binding domain, calcium-binding domains, cleavage sites for matrix metalloproteinases, and CD44 receptor-binding domain [42,43,44,45,46,47,48,49]. Five OPN splice variants have been identified. OPNa (full-length isoform) contains all seven exons while OPNb lacks exon 5 and OPNc lacks exon 4 (Figure 1). OPN4 lacks exons 4 and 5 and OPN5 contains all seven exons plus an extra exon (yields alternative translation start) between the third and fourth exons [50]. It remains to be determined whether OPN4 and OPN5 transcripts are translated to protein. Unique phosphorylation sites and transglutaminase cross-linking sites allow for structural and functional differences between isoforms [48]. Differential expression of OPN isoforms is likely to contribute to cancer-specific progression. For example, OPNc supports anchorage independence and invasiveness in breast cancer while OPNa and OPNb promote migration in hepatocellular carcinoma [51,52,53].

## 3. OPN Receptors

**Figure 2:**
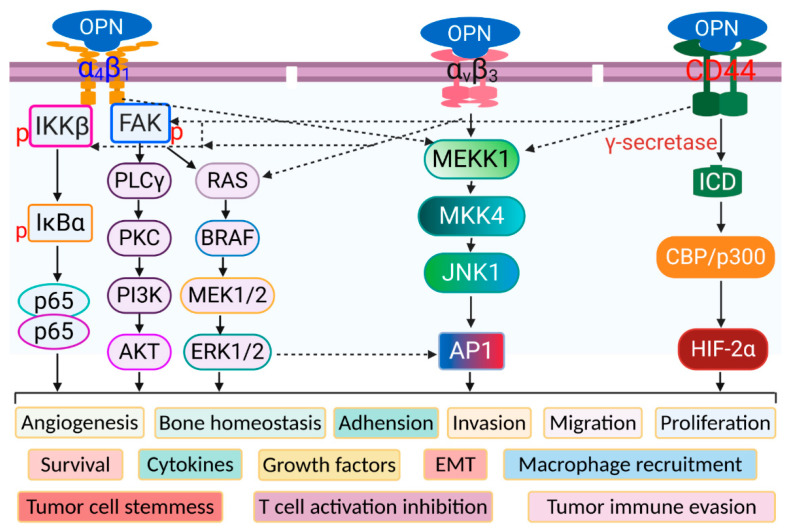
OPN receptors and their signaling pathways. The three major OPN receptor-interacting domains binds to their respective receptors to activate distinct and overlapping signaling pathways, which regulate cellular processes under physiological conditions and disease progression under pathological conditions such as cancer.

Integrins and CD44 receptors are among OPN’s known binding partners and activate signal cascades exploited by OPN in the process of cancer progression (Figure 2). Integrins allow cells to bind extracellular matrix and transduce cellular signals. The CD44 family of receptors regulates migration, invasion, angiogenesis, and metastasis [54]. Blocking OPN’s ligation with these receptors is a focus of ongoing extensive research area.

### 3.1. CD44 Receptor

CD44 is a ubiquitously expressed surface glycoprotein with three domains. The extracellular domain interacts with the extracellular environment, and the transmembrane domain allows for interactions with adaptor proteins, and the intracellular domain acts as a transcription factor [55]. CD44 has multiple variant isoforms generated by alternative splicing of exons. Standard CD44 (CD44s) is the smallest of the CD44 isoforms as it lacks the entire variable region present on the variant isoforms. CD44s was originally isolated from lymphoid cells where it was involved in endothelial cell recognition and lymphocyte homing [56]. However, CD44s has now been found in a variety of tissues and is considered ubiquitous. By comparison, variant CD44 isoforms are expressed principally by epithelial cells and leukocytes [57]. CD44 is involved in a variety of cellular processes. These include cellular adhesion, lymphocyte activation, lymphocyte homing, angiogenesis, wound healing, and hematopoiesis [56,58,59,60,61,62,63,64,65,66].

The involvement of CD44 in metastasis has been reported in colorectal cancer, prostate cancer, breast cancer, lung cancer, bladder cancer, melanoma, and pancreatic cancer [67,68,69,70,71,72,73]. The ability of CD44 to bind to extracellular ligands such as OPN, hyaluronic acid, and matrix metalloproteinases allows for cellular anchoring and activation of specific signaling pathways and matrix metalloproteinases. In breast cancer, the interaction between CD44 and hyaluronic acid was found to be protective, but in the other previously mentioned cancers, CD44 promoted metastasis. This supports the complex role of CD44 in cancer progression. CD44 usually promotes progression through metastasis, but there are cases where it may serve to anchor the cancer cells to the extracellular matrix and abolishing that ability would likely be detrimental.

OPN signaling via CD44 was found to promote aggressive glioma growth and stem cell phenotypes [74]. Supporting CD44’s role in glioma aggressiveness, CD44^+/+^ tumors were the most aggressive, CD44^−/−^ tumors were the least aggressive, and CD44^−/+^ tumors were somewhere in between. CD44 and OPN were found to colocalize in the perivascular niche (where stem-like glioma cells reside), so the question of whether OPN activated CD44 to cause a stem-like phenotype was asked. Indeed, OPN-treated PDGF-induced glioma primary cultures (PIGPCs) showed increased stem cell markers. When PIGPCs were treated with a gamma-secretase (protein responsible for cleaving CD44 ICD) inhibitor, the positive effect of OPN on stem cell markers was inhibited, suggesting that OPN exerts its regulation of stemness through the intracellular domain of CD44. Further supporting this claim, expression of CD44 ICD was able to induce stem cell markers in PIGPCs, primary human glioblastoma multiforme cells, and a malignant glioblastoma cell line. CD44 ICD exerted these stemness-promoting effects through enhancing HIF-2α (as supported by the results of HIF-2α knockdown experiments). The enhancement of HIF-2α activity was shown to be dependent on CBP/p300. Taken together, these data suggest that OPN binding with CD44 in gliomas initiates a stem-promoting cascade that begins with the cleavage of CD44 ICD and results in the transcription of stem cell genes mediated by HIF-2α and CBP/p300.

OPN signaling via CD44 has been implicated in the metastasis of bladder cancer cells. As was studied by Ahmed et al., tumor-associated macrophages release OPN which binds to the receptor, CD44 [75]. TIAM1 is a guanine exchange factor for Rac1 and is activated upon binding with the intracellular domain of CD44. Activated Rac1 is involved in the induction of DNA synthesis, actin cytoskeletal rearrangement, and cell migration [76]. Rac1 activation by TIAM1 is an important component of the OPN-CD44-TIAM1-Rac1-mediated mechanism of invasion in bladder cancer cell lines [75].

In colorectal cancer, OPN binding to CD44v6 promoted clonogenicity of cancer cells. In vitro, OPN neutralization abolished clonogenicity in these cancer cells. When a CD44v6-blocking antibody was administered, JNK activation was reduced. Taken together, this suggests that OPN binds to CD44v6 and promotes clonogenicity through activation of JNK [77]. Ligation of CD44v6/v7 by OPN was also investigated in gastric adenocarcinoma cells and colorectal cancer cells and found to enhance survival through activation of integrin signaling [78]. This highlights the importance of CD44 receptors in cancer while also calling attention to the complexity of OPN’s signal transduction pathway.

### 3.2. Integrin Receptors

Integrin receptors have been implicated in metastasis of breast cancer, colorectal cancer, lung cancer, prostate cancer, melanoma, and ovarian cancer [79,80,81,82,83,84]. In lung cancer and breast cancer, integrin expression has been associated with poor prognosis [85,86,87,88,89]. In colon cancer, one study found that low integrin expression was an indicator of poor prognosis [90]. OPN binds to αvβ1, αvβ3, αvβ5, αvβ6, α8β1, and α5β1 integrins through the RGD domain and α9β1, α4β1, α4β7 integrins through the SVVYGLR domain [91].

OPN secreted from pancreatic stellate cells (PSC) was shown to function through activation of integrin αvβ3 to induce epithelial to mesenchymal transition (EMT) and cancer stem cell (CSC) properties in a pancreatic cancer cell line [92]. OPN expression was determined to be higher in PSCs under hypoxic conditions rather than under normoxic conditions. When hypoxia was reversed, OPN production decreased, thereby implicating hypoxia in the expression of OPN. When pancreatic cancer cells were cultured with PSC-cultured medium supernatant, CSC-like traits were increased (as indicated by increased tumorsphere formation) and EMT markers were upregulated. The effects of PSC medium on EMT and CSC were hindered when OPN-neutralizing antibodies were introduced, confirming that OPN is responsible for the EMT and CSC. Similarly, αvβ3-neutralizing antibodies also attenuated the effects of OPN on EMT and CSC indicating the importance of αvβ3 on the studied effects. An agent inhibiting protein FOXM1 decreased the effects of OPN. Akt and ERK inhibitors inhibited the increase in FOXM1 caused by OPN-αvβ3. Taken together, these data suggest that hypoxia-induced OPN secreted from PSCs binds to integrin αvβ3 on pancreatic cancer cells. The ligation of OPN with this integrin causes a downstream increase in the expression of FOXM1 through an Akt/Erk-dependent pathway leading to EMT and increased CSC properties in pancreatic cancer cells.

OPN promotes a CSC-like phenotype in hepatocellular carcinoma cells through binding with αvβ3 integrin and subsequent downstream involvement of NF-κB and HIF-1α. In cells overexpressing OPN, both NF-κB activity and HIF-1α promoter activation was increased. An NF-κB inhibitor in cells treated with OPN resulted in a reduction of activation at the HIF-1α promoter. These results suggest that OPN-induced HIF-1α expression is regulated by NF-κB. In other words, NF-κB comes first in the pathway and HIF-1α expression follows. Indeed, ChIP analysis supported the interaction of NF-κB with the HIF-1α promoter. The blockage of the αvβ3 integrin inhibited the activation of both NF-κB and the HIF-1α promoter, suggesting αvβ3 as an upstream receptor in the pathway. The blockage of this receptor resulted in a decrease in the CSC-like property of sphere formation. Taken together, these data suggest that OPN binds to integrin αvβ3 and activates NF-κB. NF-κB then upregulates HIF-1α expression leading to the stem-like phenotype [93].

## 4. OPN and Cancer

OPN has been widely implicated in cancer invasion and metastasis, and its expression is associated with poor prognosis in a wide variety of cancers. As such, it is emerging as an important cancer biomarker and its relevance in PI3K/Akt and integrin-NF-κB/HIF-1α pathways is currently a focus of ongoing research [93,94,95,96,97,98]. OPN expression in the context of cancer includes regulation by hyaluronic acid, IL-6, transmembrane protein TM4SF4, and Oct4-Egr1 pathway [94,99,100,101]. Angiogenesis, degradation of ECM, the formation of lamellipodia, and switching phenotypes towards either cancer-associated fibroblasts or stem cells are ways in which OPN can promote invasion and metastasis. In lung, breast, colorectal, and head and neck cancer, OPN has been implicated in treatment resistance [36,102,103,104,105,106]. The following sections focus on the involvement of OPN in specific cancer types.

### 4.1. Glioma

Gliomas are malignant tumors arising from glial components of the nervous system, out of which glioblastomas are the most malignant. Increased OPN expression is correlated with poor patient survival, particularly in glioblastomas. Cytoplasmic OPN in glioblastoma biopsies, high OPN in serum from glioblastoma patients, and high OPN in plasma from glioblastoma patients were all associated with poor patient prognosis [14,15,16].

According to the “go or grow” phenomenon discovered in astrocytoma cells, a cancer cell has two options [107]. If conditions are favorable, it will stay and “grow”. If conditions are unfavorable, the cancer cell must “go” to a new environment that is more suitable. This ability to “go” can have deadly consequences for the host, because it puts them at risk of metastasis or tissue invasion. The ability of glioma cells to migrate and invade is being explored in the context of OPN, and most studies point to OPN playing a pro-migratory/invasive role. The role of OPN on invasiveness and tumor growth was studied in two glioma cell lines with different levels of endogenous OPN production [108]. In the highly invasive, OPN-overexpressing glioma cell line U87MG, when OPN was knocked down, invasive capabilities (as measured by matrigel transwell migration) dropped. To validate this in vivo, mice were implanted with a U87MG cell line with either silenced OPN or a control vector. Mice with the OPN-silenced implantations had smaller tumor size compared with controls. Another cell line, GBM-SKH, was used to examine OPN’s effects on glioma invasion. This cell line had much lower endogenous OPN and lower invasive capabilities. When OPN was overexpressed in these GBM-SKH cells, invasive capabilities were enhanced. In vivo, mice implanted with OPN-overexpressing GBM-SKH cells showed larger tumors than controls. In each experiment, the production of OPN either endogenously or through overexpression promoted invasiveness in vitro and ultimately increased tumor growth in vivo.

Some mechanisms by which glioma cells increase movement depend on OPN. Hyaluronic acid (HA) was found to induce OPN expression causing a subsequent enhancement in glioma cell motility [94]. In PTEN-deficient glioma cells, transient expression of wild type PTEN inhibited HA-induced OPN expression. This strongly suggests that PTEN plays a protective role against HA-induced OPN expression and that its mutation can contribute to OPN overproduction. Also investigating the mechanism by which HA leads to OPN overproduction, when PI3K, Akt, and mTOR were inhibited, the HA-induced expression of OPN was also inhibited. This indicates that PI3K, Akt, and mTOR are necessary for HA-induced OPN expression. Furthermore, OPN neutralization decreased HA-dependent cell motility (assessed via Boyden chamber) while the addition of rhOPN increased it. Taken together, these data support that OPN, through HA-induction, promotes glioma cell motility. While this experiment indicates that glioma cells are capable of producing OPN, normal brain-derived OPN should not be overlooked in the progression of gliomas. Indeed, one study found that extracts from normal white brain matter were capable of promoting glioma cell migration [109]. Migration was significantly decreased through OPN depletion and inhibited through integrin blocking, implicating normal OPN in glioma migration.

Whereas HA appears to be an upstream regulator of OPN in gliomas, heme oxygenase 1 (HO-1) appears to be a downstream effector of OPN contributing to cell movement [110]. In a glioma cell line, OPN was found to increase HO-1 expression. To determine whether HO-1 affected OPN-induced cell migration, HO-1 inhibitors, HO-1 activators, and HO-1 siRNAs were used to modify active HO-1 levels and observe OPN’s effects on cell migration. Predictably, the inhibitor and siRNA decreased migration in response to OPN and the activator increased migration in response to OPN. The members of a glioma cell line that were able to migrate through membrane pores were selected. This subline was denoted P10 and expression of OPN and HO-1 were both found to be elevated in this cell subline. Compared with mouse implantation of the normal glioma cell line, the P10 subline exhibited infiltrative growth, tumor spread, and finger-like projections indicating that this OPN/HO-1 overexpressing subline was able to more effectively promote glioma migration in vivo. This study suggests that OPN upregulates HO-1 thereby enhancing cell migration. During glioma progression, the cell subsets with higher OPN/HO-1 expression might have a selective advantage and predominate.

One feature that can distinguish high-grade glial tumors from low-grade glial tumors is the growth of new blood vessels, typically occurring in the hypoxic tumor environment [111]. This occurs through endothelial cell proliferation, migration, and tube formation. As such, these were parameters used for measuring angiogenesis in response to glioma-derived OPN [112]. Glioma cells were modified to overexpress OPN, and endothelial progenitor cells (EPCs) were incubated in this media. Markers of proliferation (% in S phase) and migration (transwell assay) as well as tube formation were all increased in EPCs incubated in OPN-overexpressing glioma media versus controls. This indicates that glioma-derived OPN plays a role in angiogenesis. Before these results, two studies offered differing results on the effect of OPN on glioma angiogenesis. One found that high interstitial OPN in astrocytoma patients was associated with the number of newly formed blood vessels, and another found that blockade of OPN in a glioma cell line did not produce any difference in the tumor vascularization [15,113]. The location of OPN might be a factor in whether it can promote angiogenesis. When OPN was present in the media or the interstitial space, it was associated with angiogenesis. However, when it was silenced intracellularly no difference in angiogenesis was noted. Further validating extracellular OPN as a promoter of angiogenesis, OPN was found to bind to integrin αvβ3 and when αvβ3 was blocked, angiogenesis was significantly inhibited [112].

### 4.2. Hepatocellular Carcinoma

Hepatocellular carcinoma (HCC) is a type of primary liver cancer often occurring in patients with chronic liver conditions. Hepatitis B virus (HBV) and hepatitis C virus (HCV) infections often progress to HCC. This is thought to occur, at least partly, through cirrhosis of the liver. It has been shown that OPN is upregulated in the plasma of patients with HCC versus patients with HCV-related liver cirrhosis or patients with chronic HCV and HBV infections indicating that OPN may offer a potential way to monitor the progression from viral hepatitis to HCC [114,115]. Diagnostic ability was assessed in a large European cohort study [116]. Blood samples were taken from participants and OPN levels were measured. Within this cohort, the occurrence of HCC was recorded. A positive association between levels of OPN and risk of first HCC incidence was observed. Among those diagnosed in the first two years of follow-up, OPN performed better diagnostically than the biomarker α fetoprotein. In addition to OPN’s diagnostic potential, OPN also appears to be useful as a prognostic biomarker in HCC. High OPN expression in plasma and HCC tumors were correlated with poor patient prognosis [18,19,20,21].

HCC invasiveness is being studied in the context of OPN involvement. Demonstrating an association between OPN levels and HCC invasion, different cell lines with increasing levels of OPN production showed increasing invasive capabilities [117]. When a siRNA against OPN was used, cell migration significantly decreased. These data suggest that OPN is involved in HCC invasiveness. Another study found that forced OPN overexpression, in a weakly tumorigenic HCC cell line, did significantly enhance migration and invasion [118]. It further suggested that matrix metalloproteinase 2 (MMP-2) and urokinase plasminogen activator (both involved in extracellular matrix degradation) may be components of the invasion pathway because forced OPN overexpression resulted in their upregulation. In another investigation into gene upregulation by OPN in HCC, MMP-2 was upregulated as well as CXCR4 and its ligand SDF-1 [119]. This prompted further investigation into a possible pathway between OPN and MMP-2 mediated by CXCR4 and SDF-1. Indeed, in CXCR4 deficient cells, MMP-2 was decreased and OPN-induced invasion was decreased. These data suggest that OPN upregulates MMP-2, and thereby invasiveness, through a CXCR4/SDF-1 pathway.

EMT occurs when a polarized epithelial cell changes to acquire a more mesenchymal phenotype. This can be detrimental to cancer patients because this transition is usually accompanied by an enhanced ability to migrate and invade surrounding tissues and a decreased susceptibility to apoptosis. Biochemical markers, such as vimentin, α-smooth muscle actin, tenascin-c, are typically upregulated in EMT. A switch from E-cadherin to N-cadherin is also common in EMT. These different markers can be measured as a way to assess whether the cell population has undergone EMT.

In HCC, OPN promotes the EMT phenotype. In HCC cell lines with either high or low endogenous OPN expression, OPN was blocked using an RNA aptamer, and the effect on invasion, migration, and EMT markers were assessed. OPN blockage in the high-OPN HCC cell line exhibited decreased invasion, migration, and EMT markers, but OPN blockage in the low-OPN HCC cell line did not. This suggests that OPN promotes EMT in HCC cells. Validating this in vivo, mice implanted with high-OPN HCC cells and OPN aptamer exhibited less tumor burden and fewer EMT markers than control mice not receiving OPN aptamer [120]. Similarly, OPN was found to promote EMT and it was suggested that this might occur, in part, through stabilization of mesenchymal cytoskeletal protein, vimentin [121]. When OPN was upregulated, EMT markers increased. However, when vimentin was reduced, the OPN-induced EMT phenotype was reversed. In mice with OPN-overexpressing HCC tumors, when vimentin was silenced, tumors were smaller, and markers indicated decreased EMT. Further elucidating how OPN promotes EMT, another study suggested that protein Twist is under the control of OPN [95]. Twist was shown to promote EMT because, in a Twist KO model, EMT was reduced. Upregulation of OPN in low-OPN HCC cells increased twist while downregulation of OPN in high-OPN HCC cells did the opposite. This suggests that OPN positively regulates Twist levels. Furthermore, when a PI3K/Akt inhibitor was used, OPN-induced Twist expression was decreased. Taken together, this study suggests that OPN, through activation of the PI3K/Akt pathway, upregulates Twist. Twist then promotes EMT.

Tumor cells that have undergone EMT and acquired a mesenchymal phenotype are considered stem cells. Because OPN is involved in this transformation, it follows that OPN may be involved in the general maintenance of a stem cell-like phenotype. Indeed, evidence in HCC studies shows that this is likely the case. One study investigated OPN’s effect on stem cell markers and the mechanism by which this occurs [93]. In cells with stem-like characteristics, there was an upregulation of OPN and stem cell genes indicating an association between OPN and stemness. To determine whether OPN was an essential part of stem cell maintenance, the effects of OPN KO on different stem markers were assessed. OPN KO resulted in a decrease in different stem-like characteristics. Mechanistically, OPN was suggested to promote stemness through αvβ3/NF-κB/HIF-1α/BMI1. In OPN-depleted HCC cells, overexpression of HIF-1α and BMI1 rescued sphere formation indicating their downstream position in OPN-induced stemness. When OPN was upregulated, the activity of both NF-κB and HIF-1α increased. NF-κB inhibition in OPN-overexpressing HCC cells decreased HIF-1α activation suggesting NF-κB regulates HIF-1α activation. Taken together, these data suggest one pathway by which OPN induces stemness. Upstream of OPN, IL-6 was found to increase stemness through the upregulation of OPN [99]. Showing that IL-6 promotes stemness in HCC, IL-6 upregulation promoted the upregulation of 11 stem genes and when IL-6 was knocked down, N-cadherin, which is associated with mesenchymal stem cells, was decreased. IL-6 was found to increase OPN concentration in a dose-dependent manner. In HCC cells stimulated by IL-6, OPN-KO decreased tumorsphere formation and stem cell genes. Together, these data indicate that IL-6 is an upstream positive regulator of OPN, and, through this regulation, can promote stemness in HCC.

### 4.3. Prostate Cancer

Prostate cancer (PrC) is a slow-growing form of cancer typically arising from glandular cells of the prostate. The diagnosis of PrC is associated with advancing age. Because the risk of benign prostatic hyperplasia (BPH) also increases with age, biomarkers that differ significantly between BPH and PrC are of value. OPN has been suggested as one such biomarker. OPN was significantly higher in plasma from PrC patients compared with plasma from BPH patients and healthy donors [122]. In PrC tissue samples, OPN was higher than in both BPH and normal tissue samples [123]. High OPN levels are also correlated with poor prognosis in PrC [123,124].

Matrix metalloproteinases are enzymes that enable cells to degrade the extracellular matrix. If utilized by cancer cells, this can lead to increased tumor invasion, metastasis, and cancer progression. A correlation was noted between OPN and MMP-9 levels in PrC patients, and the levels of both proteins were correlated with increasing Gleason score and PSA levels [122]. This indicates that OPN and matrix metalloproteinase 9 (MMP-9) might interact to promote PrC progression. Forced OPN-overexpression in a PrC cell line increased MMP-9 activity in conditioned medium and cell migration when compared with controls [125]. In both OPN-overexpressing and control PrC cell lines, MMP-9 concentration was the same inside the cells indicating that OPN did not regulate intracellular MMP-9. Instead, it appeared to be more involved in the secretion and indirect activation of MMP-9. Indeed, the study found that OPN regulated the secretion of MMP-9, and that activation of MMP-9 followed CD44 expression patterns. This suggests that OPN can promote MMP-9 activation (and cell migration) through CD44 upregulation in PrC cells. Another study found confirmatory results on the effects of OPN on MMP levels and migration [126]. OPN was able to induce MMP-2 and MMP-9; and, when OPN was knocked down, MMP-2 and MMP-9 were suppressed. In the OPN KO cells, proliferation, migration, and invasion of PrC cells were reduced.

Understanding the mechanisms by which metastasis occurs is of great clinical value. OPN has been reported as significantly upregulated in the plasma of patients with PrC metastasis to bone [124]. How OPN contributes to PrC metastasis is not fully understood. OPN was found to promote metastasis of PrC by increasing fatty acid oxidation and as a result, providing tumor cells with more nutrients enabling them to migrate [127]. The loss of p62 in adipocytes increased OPN production. OPN was able to upregulate carnitine palmitoyltransferase I in PrC tumors and promote fatty acid oxidation. This suggests that OPN is a link by which a mutation in one tissue type, such as adipose, can lead to a selective advantage in another, such as PrC tumors, which ultimately leads to increased metastasis.

### 4.4. Lung Cancer

Lung cancer (LC) is the leading cause of cancer-related deaths worldwide. LC is classified into either small cell lung cancer (SCLC) or non-small cell lung cancer (NSCLC) based upon histological examination. NSCLC is more common than SCLC, but SCLC is typically more aggressive with earlier metastasis. OPN is overexpressed in LC patients [128,129]. Specifically in NSCLC patients compared with healthy controls, OPN has been reported as increased in tumor tissues and plasma samples [130,131,132]. Plasma OPN levels in NSCLC patients were reported to be higher than in patients with SCLC or healthy controls [130]. In an immunohistochemical analysis of lung tumors, OPN was higher in squamous cell carcinoma and adenocarcinoma samples (which are NSCLC subtypes) than in SCLC tumors [133]. The OPN comparison between NSCLC and SCLC indicates that OPN plays a more prominent role in NSCLC and, as a result, most studies of OPN and lung cancer have been performed on NSCLC cell lines. OPN is also associated with poor prognosis in LC patients and especially those with the NSCLC variety [27,129,131,134].

In LC, the switch between carcinoma in situ to infiltrating carcinoma is marked by an increase in invasive capabilities of the cancer cells which allows them to grow deeper into the structures of the lung and eventually metastasize. OPN appears to promote the invasion of LC cells [103,130,135]. In NSCLC lines with either high or low endogenous OPN expression, modification of these cell lines with either antibodies or forced overexpression (respectively) showed that OPN promoted NSCLC cell invasion [130]. Similarly, when OPN was silenced in NSCLC cell lines, the cells exhibited decreased invasive capabilities [103,135].

Most studies point to a pro-metastatic role for OPN in LC. Often in cancer, the first sites of metastasis are the lymph nodes. Indeed, plasma OPN overexpression was correlated with increased risk of lymph node metastasis in LC patients; OPN expression in clinical sample analyses from LC patients was correlated with lymphatic metastasis [130,135]. OPN was found to increase the migration of LC cells in an αvβ3 integrin-dependent manner, indicating a possible mechanism by which OPN can promote metastasis in vitro [136]. Mice implanted with NSCLC tumors were treated with an anti-OPN monoclonal antibody (AOM1) to neutralize OPN and observe the effects on metastasis. The growth of large metastatic lung tumors was inhibited when compared with mice given control treatment [137]. Neutralizing OPN correlated with a decrease in large metastatic tumors, but not small or medium. This study indicated that OPN neutralization decreased the growth capabilities of metastatic LC tumors. Similarly, a NSCLC cell line was transfected with OPN miRNA to silence OPN expression, and mice injected with these OPN-silenced LC cells exhibited decreased metastatic lung lesions and the lesions that occurred were smaller [138].

As the overexpression of OPN contributes to the progression of LC, understanding how OPN is regulated within the context of LC is of great value. Transcription factor Oct4 and transmembrane 4 L6 family proteins (TM4SF4) have been shown to upregulate OPN in LC. Early growth response factor 1 (Egr-1), a downstream effector of Oct4, was shown to upregulate OPN production through direct binding to the OPN promoter [139]. Oct4 was found to indirectly upregulate OPN expression through Egr1 upregulation. Unsurprisingly, Oct4 overexpressing tumors exhibited higher levels of Egr1 and OPN. Oct4-Egr1-OPN is one axis utilized by LC cells to upregulate OPN [101]. TM4SF4 promotes CSC-like properties through the upregulation of OPN. When TM4SF4 expression was suppressed with siRNA, OPN levels decreased. Similarly, forced TM4SF4 overexpression increased OPN. This data supports TM4SF4 as an upstream positive regulator of OPN. The enhanced sphere-forming ability of TM4SF4-overexpressing cells could be explained by the downstream OPN increase. When OPN was neutralized with an antibody, the enhanced sphere-forming ability was suppressed [100].

Chemotherapy and radiation resistance contribute to the low survival rate for patients with LC. Higher levels of OPN have been associated with a decreased response to platinum-based chemotherapies in LC patients [102,103,104]. One study on a SCLC cell line found that OPN enhanced chemoresistance to cisplatin through suppression of the downregulation of anti-apoptotic protein Bcl-2 and subsequent blockage of caspase-dependent apoptosis [140]. In NSCLC cells with KRAS mutation, OPN is one of the genes upregulated [141]. NSCLC cells with KRAS mutation were found to exhibit a higher degree of radiation resistance and this was determined to be, at least partly, through the upregulation of OPN. OPN depletion in KRAS mutated cells led to radiosensitization; OPN addition in KRAS wild type cells led to radiation resistance. This supports the claim that the upregulation of OPN promotes radiation resistance in NSCLC with KRAS mutations. OPN does not appear to predict radiotherapy response as a single parameter, but codetection with heat shock protein 70 allows for the integration of prognostic and predictive information that may be of value in guiding clinical decisions [142].

### 4.5. Breast Cancer

Breast cancer (BC) is the second most diagnosed cancer in women and the second leading cause of cancer death among women [143]. Most breast cancers originate in the ductal or lobular components of the breast and can be categorized as carcinoma in situ or invasive carcinoma depending on invasiveness. Other subtypes such as inflammatory and mucinous breast cancer exist but are less common. OPN was shown to be overexpressed in breast cancer tumors when compared to benign tissues [144]. While OPN was originally thought to come from tumor-infiltrating immune cells, it was later discovered that breast cancer tumor cells also produce OPN [144,145,146]. Since then, tumor OPN, plasma OPN, and serum OPN have been correlated with poor prognosis in BC patients [28,29,30,146].

Cancer-associated fibroblasts (CAFs) play a role in the proliferation, invasiveness, metastasis, and angiogenesis of cancer. When CAFs isolated from a human breast cancer tumor were injected into mice along with a breast cancer cell line, tumor growth progressed more rapidly in the mice receiving the CAF injection suggesting that CAFs can promote BC progression [147]. OPN appears to play a role in CAF-mediated BC progression. Inoculation of mice with a breast cancer cell line and either OPN-KO or OPN-wild type mouse embryonic fibroblasts showed enhanced tumor growth in mice receiving OPN-wild type fibroblasts [148]. This indicates that CAF-derived OPN promotes tumor growth. In addition, CAF-derived OPN appears to be correlated with tumor invasiveness. In invasive ductal carcinoma, CAFs showed increased cytoplasmic OPN expression. Conversely, in ductal carcinoma in situ, CAFs exhibited decreased OPN expression [149].

When MSCs differentiate into CAFs, their expression profile changes, and CAF-associated genes are upregulated. In a murine xenograft model, mesenchymal stem cells were taken from sites of BC metastasis and shown to express CAF markers in an OPN-dependent manner [150]. This suggests that the transformation from MSC to CAF is OPN dependent. OPN has been further implicated in the differentiation of CAFs. The transformation of mesenchymal stem cells into CAFs requires OPN-induced expression of cytokine TGF-β1. OPN was found to elicit dose-dependent expression of TGF-β1 in MSCs and blocking of integrin or CD44 receptors ablated this expression. When OPN signaling was blocked, the expression of CAF markers was inhibited indicating that OPN is necessary for CAF differentiation. In addition, OPN-stimulated CAF markers were ablated when TGF-β1 was silenced and restored when exogenous TGF-β1 was administered. This indicates that OPN mediates CAF transformation from MSCs by increasing TGF-β1 expression [151]. Another study found that OPN was able to reprogram normal mammary fibroblasts into CAFs, a transformation that was blocked by the addition of OPN-neutralizing antibodies [152]. The phenotypic switch was measured by the pro-inflammatory gene signature of CAFs. It was suggested that this switch happens through CD44 and αvβ3 integrin receptors. Indeed, blockage of each of these receptors resulted in specific gene differences, indicating the unique contributions to the OPN-induced CAF phenotypic shift by each receptor.

The tendency of BC to metastasize to lymph nodes, lung, liver, bone, and brain presents challenges in the treatment of BC and contributes to BC-related mortality [153]. Plasma OPN levels were correlated with poor prognosis in women with metastatic BC [30]. Suggesting that OPN promotes a metastatic phenotype, two cell lines with opposite metastatic abilities were inoculated into mice; the highly metastatic cell line was found to have high OPN expression while the low metastatic cell line was found to have low OPN expression [154]. Further highlighting OPN’s pro-metastatic role in BC, mice injected with an OPN-overexpressing BC cell line showed increased lymph node metastasis and earlier observation of lung micrometastasis than controls. Loss of RGD domain abrogated OPN-induced lymph node metastasis indicating that integrin binding might be involved in OPN-induced metastasis to specific locations [155]. Other studies further support the pro-metastatic role of OPN in BC. In a xenograft model, an aptamer against OPN significantly decreased distant metastasis in mice implanted with a BC cell line [156]. OPN downregulation and upregulation in BC cells implanted into mice resulted in either a reduction or increase in the metastatic load, respectively [157]. The altered OPN expression and subsequent change in metastasis suggest that OPN is a crucial factor in the BC metastasis pathway.

OPN is involved in promoting the chemoresistance of BC cells. In one study, Paclitaxel chemotherapy was found to activate the JNK pathway in BC cells. When JNK was inhibited, a dose-dependent reduction in OPN expression was observed, indicating that JNK activation promotes OPN expression in BC cells. Disruption of OPN was able to sensitize BC cells to multiple forms of chemotherapy used in the treatment of breast cancer. Taken together, this suggests that chemotherapy activates the JNK pathway to promote OPN-mediated chemoresistance [105]. As possible mechanisms for OPN-mediated chemoresistance, decreased activation of p53 and p38 have also been suggested [158,159].

### 4.6. Colorectal Cancer

Overexpression of OPN in CRC has been reported in tumor samples from CRC patients [22,160]. In a study using pooled sample expression profiling, OPN expression was the most consistently differentially expressed biomarker in human colon tumors compared with normal mucosal specimens. This study also found a significant correlation between OPN expression and advancing tumor stage indicating OPN’s usefulness as a biomarker of CRC progression [161]. OPN is also associated with poor prognosis in CRC patients [22,23,24].

In CRC, the most common site of distant metastasis is the liver. In tissue samples taken from CRC patients, OPN was found to be significantly elevated in hepatic metastases when compared to primary colon tumor tissues [162]. OPN was also found to be a predictive biomarker for hepatic metastasis [163]. These studies indicate that OPN may play a role in CRC hepatic metastasis. OPN downregulation decreased cell motility and invasiveness and decreased hepatic metastases after intrasplenic injection of CRC cells [164]. This suggests that OPN may promote hepatic metastasis through the enhancement of cancer cell motility and invasion in CRC. During metastasis, adhesion between identical cell types (homotypic) typically decreases and adhesion between nonidentical cell types (heterotypic) increases as the cancer cells metastasize to different locations. Indeed, CRC cell lines with forced OPN overexpression exhibited a decrease in homotypic adhesion and an increase in heterotypic adhesion indicating a possible mechanism through which OPN can increase metastasis [162]. Taken together, OPN appears to promote hepatic metastasis of CRC through enhancement of cancer cell motility and invasion and alteration of cellular interactions.

OPN is involved in the maintenance of the stem cell-like phenotype in colorectal cancer (CRC) making patients susceptible to high rates of metastasis and treatment resistance. The knockdown of OPN in CRC cells restricted the proportion of cancer stem cells as measured by the ALDH1 marker. By blocking PI3K with an antagonist, the stem cell-promoting effect of OPN was reversed, thereby implicating PI3K in the downstream effects of OPN on CRC stem cell maintenance [98]. In another study, OPN overexpression was correlated with the overexpression of stem cell marker SOX2. CRC cell lines with higher OPN expression showed higher survival rates after treatment with oxaliplatin, and the cells that were oxaliplatin-resistant showed higher levels of stem cell marker SOX2. This indicates that the OPN-induced maintenance of stem cell phenotype plays a role in CRC treatment resistance to Oxaliplatin [106].

### 4.7. Melanoma

OPN contributes to melanoma progression. Individual melanoma biopsies taken from different stages indicate that OPN expression is first acquired at the step of melanoma tissue invasion [165]. OPN expression was found to be a predictive biomarker for sentinel lymph node metastasis and burden and increased serum OPN levels correlate with melanoma metastasis to the liver [35,166]. OPN’s increase at the stage in which tumors become invasive and OPN’s association with metastasis indicates that OPN likely plays a role in different aspects of melanoma progression. Prognostically, high OPN expression is associated with reduced recurrence-free survival and disease-specific survival [34,35].

One mechanism by which OPN can promote metastasis in melanoma is through activation of MMP-2 [167]. OPN upregulation was shown to increase MMP-2 levels. OPN was also shown to induce membrane type 1 (MT1) MMP, a protein that activates pro-MMP-2 to MMP-2. To determine the mechanism by which OPN upregulated MT1 MMP, NF-κB was inhibited. Upon NF-κB’s inhibition, the OPN-induced increase in MT1 MMP was inhibited, strongly suggesting that NF-κB is an important part of the pathway. To determine if MMP-2 was responsible for the increased migration and invasion, MMP-2 expression was inhibited, and drastic suppression of OPN-induced cell migration and invasion was observed. Furthermore, anti-MMP-2 antibodies decreased the size of OPN-induced tumors indicating MMP-2’s role in tumor formation. Taken together, the data from this study suggest that OPN increases migration, invasion, and tumor formation in melanoma through activation of MMP-2 by MT1-MMP in an NF-κB dependent manner.

### 4.8. Head and Neck Cancers

OPN was found to be a negative prognostic factor for head and neck squamous cell carcinoma (HNSCC) [37]. In the laryngeal squamous cell carcinoma subtype of HNSCC, OPN levels were also correlated with poor prognosis [168]. Treatment of laryngeal squamous carcinoma cells with recombinant OPN increased proliferation and invasion, and OPN knockout prevented these effects thereby implicating OPN as a pro-invasive factor in laryngeal squamous cell carcinoma [168]. In patients with metastatic nasopharyngeal carcinoma, plasma OPN levels were significantly higher than in patients with non-metastasized nasopharyngeal carcinomas and controls, implicating OPN in nasopharyngeal carcinoma metastasis [169]. Levels of OPN in both nasopharyngeal and laryngeal carcinomas are associated with lymph node metastasis [168,170]. Collectively, these data support the role of OPN in promoting invasion and metastasis in HNSCC.

Additionally, the role of OPN in inducing tumor hypoxia and subsequent treatment resistance has been studied, although with no clear consensus. In an HNSCC cell line, OPN levels were inversely correlated with tumor oxygen levels [36]. In support of OPN’s promotion of a hypoxic environment, OPN was associated with poor outcome after radiotherapy possibly due to OPN-induced tumor hypoxia [171]. When nimorazole (an agent that sensitizes hypoxic cells to the cytotoxic effects of ionizing radiation) was given, the outcome after radiotherapy was improved. However, one study using hypoxic cell cytotoxin, TPZ, found no evidence that high plasma OPN levels were predictive of hypoxia-targeting therapy outcome [172].

### 4.9. OPN in Other Cancers

As OPN levels and its effects have been extensively studied in some cancers, other forms of cancer are just being investigated in terms of OPN involvement. OPN was found to be upregulated in the serum of pancreatic cancer patients, plasma of ovarian cancer patients, plasma from multiple myeloma patients, and in tissues from bladder cancer biopsies [32,33,173,174,175]. OPN was highly expressed in the bone marrow of patients with acute myeloid leukemia and detected in leukemia cells taken from bone marrow biopsies [38]. In acute lymphoblastic leukemia, OPN was detected in leukemia cells from all patients; however, the sample size was small [176]. In pancreatic cancer, there remains some disagreement over whether OPN is a reliable marker for distinguishing between cancer and pancreatitis. In one study, serum levels of OPN were able to distinguish pancreatic adenocarcinoma from chronic pancreatitis, but another study found that OPN serum levels were not significantly different in pancreatic cancer versus chronic pancreatitis [177,178]. If chronic pancreatitis can cause an upregulation of OPN, then the usefulness of OPN as a pancreatic cancer biomarker is limited.

Studies of OPN in ovarian cancer are centered around its usefulness as a tumor biomarker compared to the cancer antigen 125 (CA125) commonly used in the diagnosis and management of ovarian cancer. Some patients have CA125-negative breast cancer, so another biomarker would drastically improve the management of ovarian cancer in these patients. One study found that, while OPN was inferior to CA125 in predicting clinical response to therapy, OPN increased earlier in patients developing recurrent ovarian cancer [179]. OPN monitoring could allow for earlier detection of cancer recurrence than if CA125 was used alone. OPN’s inferiority in predicting treatment response dictates that, while OPN could be useful, it is not a replacement for CA125. Another study found somewhat different results [180]. In ovarian cancer patients with recurrent disease, OPN levels were not significantly different from those of healthy controls. The patients in the study had debulking surgery with oophorectomies prior to the recurrence, and so it was investigated whether OPN levels would be higher than controls when a recurrence occurred. This did not occur, and recurrent disease patients had no statistical difference in their OPN levels. Both studies provide opposing evidence on the usefulness of OPN as a way to monitor for recurrent ovarian cancer. Future studies may be able to shed light on OPN as an ovarian cancer biomarker.

OPN appears to promote angiogenesis in multiple myeloma [181,182]. It was shown that OPN production by myeloma cells was critical for angiogenesis because, in the conditioned medium of myeloma cells, OPN immunodepletion and anti-OPN antibodies blocked angiogenesis. In further support of OPN’s proangiogenic role in multiple myeloma, the study found that significantly increased bone marrow angiogenesis occurred in patients positive for OPN as compared to those negative [181]. An interaction between myeloma cells and osteoclasts mediated by OPN and VEGF was implicated in angiogenesis (as measured by vascular tubule formation) in vitro [182]. When either OPN or VEGF were independently inhibited by neutralizing antibodies, enhancement of vascular tubule formation from the osteoclast/myeloma cell-conditioned medium was partially decreased. When both OPN and VEGF were neutralized together, vascular tubule formation went down to control levels. Taken together, the data from this study indicate that OPN along with VEGF participates in an interaction between osteoclasts and myeloma cells that leads to angiogenesis.

Even though OPN’s upregulation in leukemia indicates some likely involvement, much remains unclear about how OPN contributes to leukemia. One study found that, by adhering to OPN, leukemic blasts were able to enter a dormant state thereby evading some forms of chemotherapeutics [176]. So far, a couple of studies have investigated OPN’s usefulness in prognosis [38,39]. Both have found that OPN is a negative prognostic factor for patients with acute myeloid leukemia.

## 5. OPN Function as an Immune Checkpoint in the Tumor Microenvironment

OPN was originally identified as an immune regulatory molecule in activated T cells and was initially termed the early T cell activation gene (Eta-1) [7]. The expression and function of OPN in immune cells have since been extended to B cells, NK cells, NKT cells, macrophages, dendritic cells, monocytes, neutrophils, and eosinophils [9,183,184,185]. Under physiological conditions, OPN regulates host immune response against infection via enhancing Th1 and Th17 polarization by inducing hypomethylation of *IFNG* and *IL17a* [185]. Furthermore, OPN has been shown to regulate IL6 and IL12, and downregulate IL10 expression in monocytes, repress IL27 expression in dendritic cells, and acts as chemoattractant cytokine for recruitment of macrophages and neutrophils [185,186,187]. Under pathological conditions, OPN has been implicated in immune cell-mediated inflammatory diseases, including lupus erythematosus, multiple sclerosis, rheumatoid arthritis, intestinal bowel disease, type I diabetes, and Sjögren’s syndrome [185,187,188]. Although the immune regulatory functions of OPN have been proven in various inflammatory and autoimmune disease models, the involvement of and mechanism underlying OPN function in the tumor microenvironment is still incompletely understood [187].

### 5.1. OPN Promotes Tumor Development through Recruitment of Macrophages and Suppression of T Cell Activation

Emerging experimental data indicate that OPN functions in the tumor microenvironment through regulating macrophages and T cells [189,190]. Immunosuppressive actions of OPN on macrophages include M2 polarization, cancer cell chemoattraction, and increased COX-2 expression. OPN also appears to suppress T cell activity in the tumor environment. Additionally, OPN regulates PD-L1 expression in macrophages further contributing to cancer immunosuppression.

M2 macrophages were found to significantly increase upon treatment of monocytes with OPN-positive conditioned medium from gastric cancer cells when compared to monocytes that were treated with an OPN-deficient media from gastric cancer cells [191]. This suggests that OPN from cancer cells promotes M2 polarization. Furthermore, mice xenografted with OPN-positive gastric cancer cells and monocytes exhibited faster tumor growth with poorer survival than in controls with monocytes and OPN-silenced cancer cells. However, not all literature supports the claim that OPN promotes M2 polarization. A more recent study utilized monocytes from healthy donors and incubated them with different concentrations of recombinant OPN. OPN did not increase the amount of M2 macrophages because no significant change was observed in any of the M2 macrophage markers tested [190]. Instead, it was suggested that OPN was more involved in the maintenance of the M2 phenotype.

TAMs produce OPN within the tumor microenvironment which contributes to cancer progression. In SCLC, TAM-produced OPN (TOPN) was found to be a negative prognostic factor [192]. In one study, macrophages cocultured with patient-derived CD44^+^ CRC cells exhibited increased production of OPN. This highlights the ability of cancer cells to upregulate OPN production in macrophages. CRC cells from patient tumor samples were inoculated into mice alone or with TAMs. When the xenograft tumors were excised, the TAM-inoculated tumors exhibited increased OPN in the tumor stroma and tumor island [77]. Additionally, media from CRC cells cocultured with monocytes was able to enhance the clonogenicity of multiple CRC cell lines isolated from patients when compared to media from CRC cells alone. When OPN was depleted with a neutralizing antibody, the clonogenicity promoted by the CRC cell + monocyte media was abolished [77]. Taken together, this indicates that cancer cells can upregulate OPN production in macrophages and that the secreted OPN plays a role in enhancing the clonogenicity of cancer cells.

While TAMs produce OPN, OPN also affects the migration of TAMs. In further support of the chemoattractant capabilities of OPN on TAMs, another study found that OPN-KO decreased the infiltration of macrophages into tumor tissue and that OPN KO had no effect on the infiltration of macrophages into normal tissues [193]. This indicates that OPN regulates the infiltration of macrophages into tumor tissue, specifically.

OPN-deficient mice with gliomas exhibited prolonged survival due to an increase in T cell effector activity and a decrease in macrophage infiltration [190]. The frequency of IFNγ, TNF-α, and IL-12 producing T cells was decreased in samples from the spleen, blood, and/or brain of OPN-deficient mice, indicating that OPN suppresses the activity of effector T cells. Further supporting OPN’s immunosuppressive role in glioma-bearing mice, immune-suppressive regulatory T cells were decreased in the blood of OPN-deficient mice. Another study found that, when macrophages were cocultured with a cancer cell line, there was a decrease in CD4^+^ T cell activity. However, OPN inhibition rescued CD4^+^ T cell activity [194].

OPN also appears to promote the M2 polarization of microglia (a specialized population of macrophages found in the central nervous system). OPN in the glioma microenvironment can undergo sequential proteolytic processing by thrombin and MMP-3 and/or MMP-7. This tumor-derived processed form of OPN promotes pro-tumorigenic microglia via integrin-mediated activation of the FAK/Akt pathway to induce M2 reprogramming. However, when OPN came from non-transformed cells, the M1 phenotype was induced in microglia implicating tumor-associated proteolytic processing of OPN as a possible mechanism for differential microglia programming [195].

In melanoma, the expression of COX-2 by macrophages promotes tumor growth through the promotion of angiogenesis and migration of cancer cells. The percentage of COX-2 positive TAMs can act as a biomarker for melanoma progression [196]. Interestingly, OPN was found to induce COX-2 expression in macrophages [193]. Mechanistically, ERK and p38 inhibitor experiments suggested that OPN-induced COX-2 expression occurs through an ERK/p38 dependent mechanism. OPN-stimulated macrophages were found to increase the angiogenic response in HUVEC cells, and this effect was blocked upon the addition of a COX-2 inhibitor. Similarly, OPN-stimulated macrophages were found to promote melanoma cell migration, but upon the addition of a COX-2 inhibitor, migration was also blocked. This indicates that OPN-stimulated macrophages promote melanoma angiogenesis and cell migration in a COX-2 dependent manner [193].

The expression of PD-L1 by either cancer cells or macrophages allows for immune evasion through immunosuppressive interactions with tumor-specific T cells [197]. In HCC, OPN was found to promote PD-L1 expression through activation of the CSF1-CSF1R pathway in macrophages. Blocking PD-L1 and CSF1R resulted in higher CD8^+^ T cell infiltration [198]. Another study found that in OPN-deficient macrophages, PD-L1 was downregulated, thereby supporting OPN’s role in the expression of PD-L1. Upon macrophage PD-L1 inhibition, M1 markers began to predominate over M2 markers. This indicates that PD-L1 expression can promote the immunosuppressive M2 phenotype of macrophages [194].

### 5.2. OPN Is Highly Expressed in Myeloid-Derived Suppressor Cells and Acts as an Immune Checkpoint That Bridges Innate Immune Cells to T Cells in the Tumor Microenvironment

Myeloid-derived suppressor cells (MDSCs) are a heterogeneous population of immature myeloid cells commonly present in cancer [199]. They have been found to inhibit both adaptive and innate immune systems by altering the signaling, migration, and environment of T cells and through cell-to-cell contact with natural killer cells [200]. MDSCs are classified as either granulocytic or monocytic depending on the differential expression of specific surface epitopes, Ly6G or Ly6C, respectively. In tumors, granulocytic MDSCs predominate [201]. Downregulation of interferon regulatory factor-8 (IRF-8) has emerged as a tumor mediated event that can lead to the accumulation of MDSCs [202].

One of the hallmarks of IRF8 KO mice is the massive accumulation of CD11b^+^Gr1^+^ immature myeloid cells that phenotypically and functionally resemble MDSCs [202,203]. CD11b^+^Gr1^+^ cells from IRF8 KO mice have a drastically higher level of OPN expression level as compared to the CD11b^+^Gr1^+^ cells in WT mice suggesting that IRF-8 negatively regulates the expression of OPN [189]. Further analysis revealed that the CD11b^+^Ly6G^+^Ly6C^−^ cells, or granulocytic MDSCs, are the subset of myeloid cells that express elevated OPN. Similar to what was observed in IRF8 KO mice, OPN is also highly expressed in the CD11b^+^Ly6G^+^Ly6C^−^ PMN-MDSCs of tumor-bearing mice. Mechanistically, IRF8 was found to directly bind to the Spp1 promoter to repress OPN expression in MDSCs. Functionally, recombinant OPN protein inhibits both mouse and human T cell IFNγ production and proliferation T cell IFNγ is an anti-tumorigenic cytokine that can directly enhance the motility and cytotoxicity of CD8^+^ T cells [189,204]. As such, inhibition of IFNγ through OPN would promote the immunosuppressive tumor environment. Because IRF8 is often silenced in tumor cells and MDSCs, it appears that tumor cells and MDSCs use silencing IRF8 as a mechanism to induce OPN expression to suppress T cell activation to promote tumor progression, which provides a strong rationale for neutralizing OPN in cancer patients to suppress tumor immune escape [202,203,205,206].

### 5.3. OPN Neutralization Monoclonal Antibody for Cancer Therapy and Immunotherapy

Neutralization of OPN has therapeutic potential for cancer treatment and has been shown to lessen the severity of multiple inflammation-mediated diseases, including osteoporosis, hepatitis, and arthritis (Table 1). Some monoclonal antibodies have been studied in the context of cancer and others have not. In the future, OPN neutralizing cancer studies may investigate antibodies that, as of now, have only been investigated in terms of inflammation-mediated diseases.

An anti-OPN monoclonal antibody 23C3 was shown to decrease the bone loss associated with oophorectomies [207]. Mice treated with 23C3 mAb showed a decreased loss of tibial cortical and trabecular mass after oophorectomies. An earlier study on OPN neutralization in collagen-induced arthritis found that 23C3 reduced pro-inflammatory cytokines (many of which induce osteoclast differentiation and function) and promoted the apoptosis of type-II collagen activated T cells, thereby indicating a mechanism by which OPN neutralization by 23C3 improves arthritis and osteoporosis [208]. A humanized version of 23C3, Hu23C3, was also shown to suppress the development of hepatitis in mice injected with Concavalin A [209]. Another anti-OPN mAb, C2K1, was able to decrease collagen-induced arthritis in a primate model. After the induction of arthritis in Cynologus monkeys, C2K1 antibodies were administered and C2K1 levels were periodically measured. Plasma levels of C2K1 in the blood were inversely correlated with the degree of joint swelling compared to a more stable level of joint swelling in the control group. Additionally, histological analysis showed less bone erosion and destruction, less cartilage degeneration, and less pannus formation in monkeys treated with C2K1. This indicates that the neutralization of OPN by mAb CK21 may decrease the severity of arthritis [210].

The OPN level is elevated in serum and correlated with poor prognosis in several types of human cancers, providing a strong rationale for neutralizing OPN in human cancer therapy and immunotherapy [16,211,212,213,214]. AOM1 is an anti-OPN mAb that blocks the integrin αvβ3 binding site and the thrombin cleavage site on OPN. One study showed that human tumor cells from specific cancers and monocytes (all of which expressed αvβ3 receptors) migrated towards OPN. When AOM1 was introduced, migration was inhibited [137]. This suggests that the AOM1 blockage of the integrin-binding site on OPN is sufficient to inhibit migration. Additionally, treatment of NSCLC tumor-bearing mice with AOM1 showed an inhibition in the growth of pre-seeded metastatic lesions. Because primary tumor growth was not affected by AOM1, this suggests that OPN neutralization may be more effective in inhibiting metastatic growth than primary growth. Another anti-OPN mAb targeting the SVVYGLR motif of OPN was found to significantly inhibit tumor growth in adult T cell leukemia tumor-bearing mice [215]. This antibody also suppressed the metastasis of subcutaneously inoculated adult T cell leukemia cells into peripheral blood and liver tissues and reduced the number of cancer-associated fibroblasts in primary tumor tissues. Neutralization of OPN with mAb MPIIIB10 was shown to delay tumor growth in mice injected with a colon cancer cell line. Additionally, when MPIIIB10 was given with a B-cell based vaccine, there was delayed tumor growth when compared with a B-cell based vaccine given alone. This suggests that OPN neutralization offers some benefit when given alone but is also synergistic when given with immunotherapeutic vaccines [216].

Although studies have demonstrated the concept of OPN neutralization, the feasibility of the strategy warrants consideration. In one study aimed at answering this exact question, the results show that drastic OPN neutralization may be difficult [217]. As calculated from serum samples in three healthy subjects, OPN was determined to have a very fast turnover rate with an average half-life of 11 min. Using pharmacokinetic and pharmacodynamic modeling of a standard antibody, a 20% reduction in free OPN concentration would require high doses given at weekly intervals. However, if a modified sweeper antibody was used in high, weekly doses, OPN knockdown of 90% might be achievable. One clinical trial assessed the effects of humanized mAb, ASK8007, on rheumatoid arthritis in patients [218]. It was safe even at high doses but did not show any treatment efficacy. It is possible that, due to the short half-life of OPN, the mAb was not able to achieve sufficient target coverage. Frequent administrations of large doses might be challenging in the implementation of anti-OPN treatments, but new developments in antibody technology are promising. In the context of human cancer, reducing OPN to the physiological level could be an effective therapeutic and immunotherapeutic approach. Future research may provide more insight into the efficacy and feasibility of OPN neutralization.

## 6. Conclusions

**Figure 3:**
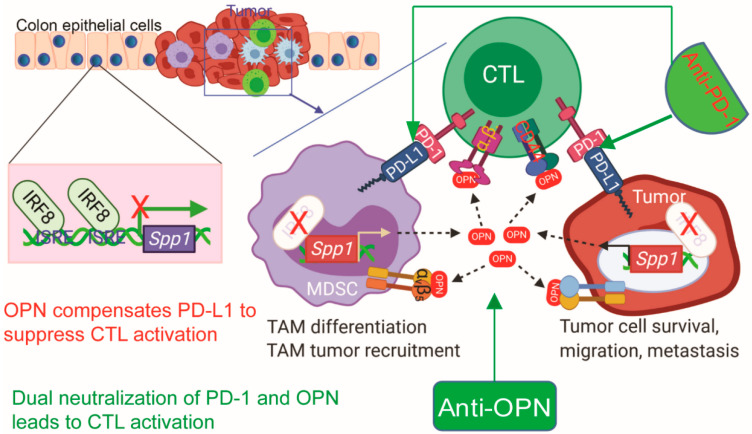
OPN bypasses Anti-PD-1 immunotherapy to suppress T cell activation. OPN suppresses T cell activation and renders Anti-PD-1 immunotherapy less efficacious. Neutralizing OPN may allow patients to overcome resistance to current ICB immunotherapy.

Recent single-cell RNA sequencing revealed that OPN is primarily expressed in tumor cells and tumor-infiltrating myeloid cells in human breast and colorectal cancer patients [225,226]. Among the myeloid cell populations, we have recently determined that OPN is highly expressed in the polymorphonuclear myeloid-derived suppressor cells (PMN-MDSCs). As depicted in Figure 3, OPN acts as another immune checkpoint that negatively regulates T cell activation, which may render PD-1-based immune checkpoint blockade (ICB) immunotherapy less efficacious and promote patient non-response to ICB therapy [189]. Therefore, OPN blockade immunotherapy with OPN neutralization mAbs may represent an effective approach to increase the efficacy of PD-1-based ICB immunotherapy in responding patients and to overcome resistance to ICB immunotherapy in non-responding patients. Because OPN is involved in adaptive processes such as healing, safety concerns regarding OPN neutralization are warranted [227,228]. However, during OPN neutralization for the treatment of rheumatoid arthritis adverse events such as nausea, bronchitis, dizziness, rash, and myalgia were reported but did not differ significantly from the placebo group [218]. There are limited clinical trials assessing the safety of OPN neutralization, but at this time it does not appear to be associated with significant adverse effects. Current available OPN neutralization mAbs were not developed for blocking OPN function in suppression of T cell action. Considering the multiple receptor-binding domains in OPN and the many different receptors and respective signaling pathways, it is thus critically important to develop an OPN neutralization mAb that specifically blocks OPN function in suppression of T cell activation.

## Figures and Tables

**Table 1:** OPN-neutralizing monoclonal antibodies.

Name	Antigen	Isotype	Host	Disease	Notes	Ref
23C3	WL_PDP sequence on OPN	IgG	Mouse	Arthritis	Development of arthritis inhibited, progression of arthritis significantly reversed, reduction of proinflammatory cytokines, enhanced apoptosis of activated T cells	[208]
				Osteoporosis	Reduced loss of cortical and trabecular bone mass, decreased number and area of osteoclast-mediated bone pits, promotion of osteoclast apoptosis	[207]
				Hepatitis	Protective against liver damage in hepatitis mouse models, inhibition of monocyte migration, inhibition of T and NKT cell infiltration, reduction of proinflammatory cytokines	[209]
				APAP toxicity	Pretreatment effectively prevented APAP-induced hepatotoxicity through inhibition of inflammatory infiltration	[219]
AOM1	SVVYGLRSKS sequence immediately adjacent to the RGD motif of human OPN	IgG2	Phage display libraries led to discovery of fully human mAb	NSCLC	Reduced cell migration in vitro, and decreased the number of large metastatic lung tumors in mice	[137]
MPIIIB10	Rat OPN	IgG1	Mouse	Cardiac remodeling	Partially blocked Angiotensin II-induced collagen gel contraction by cardiac fibroblasts	[220]
				Glomerulonephritis	Reduced glomerular and tubulointerstitial infiltration of macrophages/monocytes	[221]
2C5	Recognizes epitope in mouse OPN that is near the amino terminal side of the RGD integrin binding site	IgG1	Mouse	Stress-induced thymus atrophy	Ameliorates stress-induced thymus atrophy in mice	[222]
10A16	Human OPN	IgG1	Mouse	Tuberculosis	Reduced the amounts of IL-12 and IFNγ produced from *M. Bovis* infected peripheral blood mononuclear cells	[223]
C2K1	SVVYGLR of human OPN	2K1 mAb fused to human IgG1	Chinese hamster	Arthritis	Inhibition of joint swelling (corresponding to mAb blood levels) and prevention of bone and cartilage destruction in a primate model of collagen-induced arthritis	[210]
Humanized 1A12	NAPSD motif adjacent to the calcium binding domain on OPN	IgG1	Mouse	Breast cancer	Inhibited cell adhesion, migration, invasion, and colony formation in a breast cancer cell line, suppressed primary tumor growth and spontaneous metastasis	[224]
N/A	SVVYGLR of mouse OPN	IgG	Mouse	Adult T cell Leukemia	Inhibited tumor growth, invasion, and metastasis in ATL mice models, reduced the number of fibroblast activating protein positive fibroblasts	[215]
ASK8007	SVVYGLR	IgG1	Humanized	Rheumatoid arthritis	Safety and efficacy of OPN neutralization assessed in clinical trial, no apparent clinical responses *	[218]

* Clinical trial. All others preclinical studies.
